# Association of ADAMTS proteoglycanases downregulation with IVF-ET outcomes in patients with polycystic ovary syndrome: a systematic review and meta-analysis

**DOI:** 10.1186/s12958-022-01035-9

**Published:** 2022-12-13

**Authors:** Yanbin Shi, Yang Shi, Guiyuan He, Guang Wang, Hongbo Liu, Xiaoguang Shao

**Affiliations:** 1 School of Public Health, China Medical University, Shenyang, China; 2 Reproductive and Genetic Medicine Center, Dalian Women and Children’s Medical Center, Dalian, China

## Abstract

**Background:**

A disintegrin and metalloproteinase with thrombospondin-like motifs (ADAMTS) is involved in inflammation and fertility in women with polycystic ovary syndrome (PCOS). This study aims to assess the role of ADAMTS level in the outcomes of in vitro fertilization and embryo transfer (IVF-ET) in women with PCOS, using a meta-analytic approach.

**Methods:**

We systematically searched Web of Science, PubMed, EmBase, and the Cochrane library to identify potentially eligible studies from inception until December 2021. Study assess the role of ADAMTS levels in patients with PCOS was eligible in this study. The pooled effect estimates for the association between ADAMTS level and IVF-ET outcomes were calculated using the random-effects model.

**Results:**

Five studies involving a total of 181 patients, were selected for final analysis. We noted that ADAMTS-1 levels were positively correlated to oocyte maturity (*r* = 0.67; *P* = 0.004), oocyte recovery (*r* = 0.74; *P* = 0.006), and fertilization (*r* = 0.46; *P* = 0.041) rates. Moreover, ADAMTS-4 levels were positively correlated to oocyte recovery (*r* = 0.91; *P* = 0.001), and fertilization (*r* = 0.85; *P* = 0.017) rates. Furthermore, downregulation of ADAMTS-1, ADAMTS-4, ADAMTS-5, and ADAMTS-9 was associated with elevated follicle puncture (ADAMTS-1: weighted mean difference [WMD], 7.24, *P* < 0.001; ADAMTS-4: WMD, 7.20, *P* < 0.001; ADAMTS-5: WMD, 7.20, *P* < 0.001; ADAMTS-9: WMD, 6.38, *P* < 0.001), oocytes retrieval (ADAMTS-1: WMD, 1.61, *P* < 0.001; ADAMTS-4: WMD, 3.63, *P* = 0.004; ADAMTS-5: WMD, 3.63, *P* = 0.004; ADAMTS-9: WMD, 3.20, *P* = 0.006), and Germinal vesicle oocytes levels (ADAMTS-1: WMD, 2.89, *P* < 0.001; ADAMTS-4: WMD, 2.19, *P* < 0.001; ADAMTS-5: WMD, 2.19, *P* < 0.001; ADAMTS-9: WMD, 2.89, *P* < 0.001). Finally, the oocytes recovery rate, oocyte maturity rate, fertilization rate, cleavage rate, good-quality embryos rate, blastocyst formation rate, and clinical pregnancy rate were not affected by the downregulation of ADAMTS-1, ADAMTS-4, ADAMTS-5, and ADAMTS-9 (*P* > 0.05).

**Conclusions:**

This study found that the outcomes of IVF-EF in patients with PCOS could be affected by ADAMTS-1 and ADAMTS-4; further large-scale prospective studies should be performed to verify these results.

## Background

Polycystic ovary syndrome (PCOS), the most common endocrine disorder among women of reproductive age, is characterized by hyperandrogenemia, hirsutism, acne, oligo-or anovulation, and polycystic ovaries [1]. The prevalence of PCOS in women of reproductive age ranges from 10 to 16% [2, 3], and it is significantly associated with infertility, obesity, insulin resistance, type 2 diabetes, dyslipidemia, cardiovascular disease, hepatic steatosis, and endometrial cancer [4–6]. The hyperresponsiveness to stimulation by luteinizing hormone and failed downregulation of thecal androgen production associated with functional ovarian hyperandrogenism, might play an important role in the pathophysiology of PCOS [7, 8]. Moreover, the insulin resistance-induced hyperinsulinemia augments the luteinizing hormone-induced homologous desensitization, thereby aggravating hyperandrogenism [9, 10]. Furthermore, follicle maturation arrest and anovulation could be caused by hyperinsulinemia, which synergizes with androgen to prematurely luteinize granulosa cells [11].

Several studies have already addressed the prognosis of PCOS [12–14]. A systematic review and found PCOS were associated with an increased risk of pregnancy-induced hypertension, pre-eclampsia, gestational diabetes and premature delivery [12]. Moreover, they point out the alteration of oocyte competence contributed an important role on subfertility for patients with PCOS [13]. Furthermore, PCOS could affect endometrium, chronic low-grade inflammation, immune dysfunction, altered uterine vascularity, abnormal endometrial gene expression and cellular abnormalities [14]. Therefore, additional potential markers for the prognosis of PCOS should be explored for the purpose of improving the prognosis of PCOS.

Several members of the a disintegrin and metalloproteinase with thrombospondin-like motifs (ADAMTS) family have been identified in growing follicles during ovulation and in the corpora lutea of several mammalian species [15–23]. These findings indicate that the members at the proteoglycanase arm of the ADAMTS family were the most expressed. However, the expression of all members of the ADAMTS family during folliculogenesis, was not systematically explored. No systematic review or meta-analysis have been conducted to assess the role of ADAMTS levels in the outcome of IVF-ET in patients with PCOS. Therefore, this study was performed to assess the potential role of members of the ADAMTS family in IVF-ET outcomes in women with PCOS.

## Methods

### Data sources, search strategy, and selection criteria

The protocol of this systematic review and meta-analysis was registered at INPLASY (ID: INPLASY202260115). The revised Preferred Reporting Items for Systematic Reviews and Meta-Analysis (PRISMA) Statement was applied to conduct and report this systematic review and meta-analysis [24]. Published articles assessing the role of ADAMTS levels in patients with PCOS, were considered eligible for this study, and all eligible articles, regardless of the language used for publication and the publication status, were included. Three databases (Web of Science, PubMed, and EmBase) and the Cochrane library, were systematically searched from their year of inception until December 2021, using the following search terms: “Polycystic ovary syndrome” [Mesh] or “polycystic ovary syndrome” or “PCOS” and “ADAMTS Protein” [Mesh] or “ADAMTS” or “Aggrecanase-1” or “A Disintegrin And Metalloproteinase With Thrombospondin Motifs”. We also reviewed the reference lists of retrieved studies to identify any new eligible study.

The process of literature search and study selection was independently performed by 2 reviewers, and conflicts between these reviewers were settled by group discussion. The inclusion criteria were as follows: (1) Patients: all patients diagnosed with PCOS; (2) Exposure: members of the ADAMTS family, including ADAMTS-1, ADAMTS-4, ADAMTS-5, ADAMTS-9, ADAMTS-19; (3) Comparison: ADAMTS level; (4) Outcomes: implantation, follicles punctured, oocytes retrieved, metaphase II oocytes, germinal vesicle oocytes, and oocyte recovery, oocyte maturity, fertilization, cleavage, good-quality embryo, blastocyst formation, and clinical pregnancy rates; and (5) Study design: no restrictions were placed on study design.

### Data collection and quality assessment

Two reviewers independently performed data abstraction and quality assessment, and disagreement between these reviewers was resolved by a third reviewer, after referring to the full text of the original article. The following information were collected from included studies: first author’s name, publication year, study design, country, sample size, mean age, body mass index (BMI), reported ADAMTS family members, and reported outcomes. The quality of observational studies was assessed using the Newcastle–Ottawa Scale (NOS), and the staring system for each study ranged from 0–9 [25].

### Statistical analysis

The associations of ADAMTS proteoglycanases level with IVF-ET outcomes in PCOS were assigned Spearman coefficients, and the pooled effect estimates were calculated using the random-effects model, which considered the underlying differences across included studies [26, 27]. The heterogeneity among included studies was assessed using *I*^*2*^ and Q statistics, and *I*^*2*^ > 50.0% or *P* < 0.10 was considered significant heterogeneity [28, 29]. The robustness of pooled conclusions was assessed using a sensitivity analysis through sequential removal of individual studies [30]. All reported *P* values for pooled results are 2-sided, and *P* < 0.05 was considered statistically significant. All statistical analysis in our study was performed using STATA software (version 10.0; Stata Corporation, College Station, TX, USA).

## Results

### Literature search

**Fig. 1:**
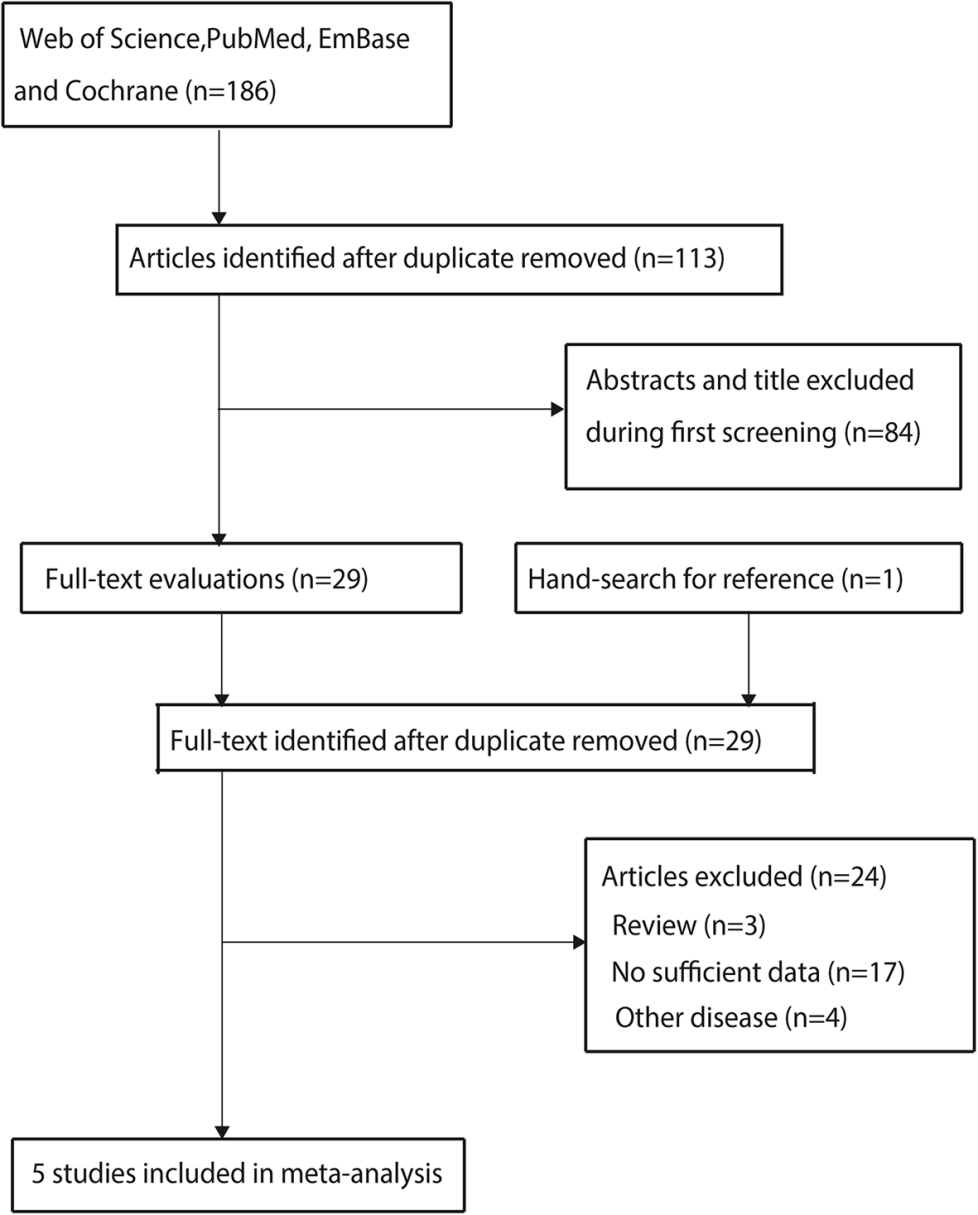
The PRISMA flowchart for the process of literature search and study selection

A total of 186 articles were identified from initial electronic searches, and 113 were retained after duplicate articles were removed. Then 84 studies were removed for having irrelevant titles or abstracts. The remaining 29 studies were selected for full-text evaluations and 24 were excluded because of Review (*n* = 3), insufficient data (*n* = 17), and other diseases (*n* = 4). The details of the literature search and study selection are shown in Fig. 1, and a total of 5 studies were included in the final quantitative analysis [31–35].

### Study characteristics

The baseline characteristics of the included studies and recruited patients are shown in Table 1. Of the 5 studies included, 3 were prospective and 2 were retrospective. One study was conducted in Turkey, 2 were conducted in Iran, and 2 were conducted in China. The mean age of included patients ranged from 28.6 to 30.5 years, while the mean BMI across included studies ranged from 22.7 to 27.9 kg/m^2^. Four studies reported on the role of ADAMTS-1, 1 reported on the role of ADAMTS-4, 1 reported on the role of ADAMTS-5, and 1 reported on the role of ADAMTS-9.

**Table 1:** The baseline characteristics of identified studies and recruited patients

Study	Study design	Country	Sample size	Age (years)	BMI (kg/m^2^)	Basal FSH (IU/L)	LH (IU/L)	LH to FSH ratio	T (ng/ml)	PRL (ng/L)	Total Gn dose (IU)	Peak E2 (pg/ml)	PG (ng/ml)	ADAMTS
Xiao 2014 [31]	Prospective	China	40	30.5	22.7	4.6	5.4	1.2	0.7	20.7	1703	3903	NA	ADAMTS-1: oocyte maturity (*r* = 0.8857; *P* = 0.0333); oocyte recovery (*r* = 0.8857; *P* = 0.0333); fertilization (*r* = 0.8313; *P* = 0.0403); cleavage rate (*r* = 0.7714; *P* = 0.1028); good quality embryo rate (*r* = 0.3714; *P* = 0.4972)
Tola 2017 [32]	Prospective	Turkey	21	29.2	27.9	7.3	6.1	NA	NA	NA	NA	NA	0.79	ADAMTS-1: metaphase II oocytes (*P* > 0.05); implantation (*P* < 0.05)
GohariTaban 2019 [33]	Prospective	Iran	37	28.6	26.5	6.0	7.1	NA	NA	NA	NA	NA	NA	ADAMTS-1: oocyte recovery (*r* = 0.39; *P* = 0.03), oocyte maturation (*r* = 0.48; *P* = 0.02), fertilization (*r* = 0.48; *P* = 0.04); ADAMTS-9: oocyte recovery (*r* = 0.78; *P* = 0.006) oocyte maturation (*r* = 0.32; *P* = 0.004); fertilization rate (*r* = 0.38; *P* = 0.07)
GohariTaban 2021 [34]	Retrospective	Iran	35	29.1	26.3	6.8	7.1	NA	NA	NA	NA	NA	NA	ADAMTS-4: oocyte recovery (*r* = 0.91; *P* < 0.001), oocyte maturation (*r* = 0.60; *P* = 0.001), and fertilization (*r* = 0.85; *P* < 0.001); ADAMTS-5: oocyte recovery (*r* = 0.54; *P* = 0.008), oocyte maturation (*r* = 0.66; *P* < 0.001), and fertilization (*r* = 0.52; *P* = 0.010)
Yang 2021 [35]	Retrospective	China	48	29.5	24.9	6.0	9.7	NA	0.47	17.68	2152	NA	0.47	ADAMTS-1: oocyte maturation (*r* = 0.3673; *P* = 0.0300), good-quality embryo (*r* = 0.3472; *P* = 0.0410), fertilization (*r* = 0.1408; *P* = 0.3993), cleavage (*r* = 0.0749; *P* = 0.6548), blastocyst formation (*r* = 0.0673; *P* = 0.7239)

^*^ *BMI* Body mass index, *E2* Estradiol, *FSH* Follicle-stimulating hormone, *Gn* gonadotropin, *LH* luteinizing hormone, *PG* Progesterone, *PRL* Prolactin, *T* Testosterone

### Quality of included studies

Table 2 summarizes the methodological quality of the included studies, and all 5 studies were of high quality (7 or more stars); 3 studies had 8 stars and 2 had 7 stars.

**Table 2:** The Newcastle–Ottawa Scale of individual study

Study	Selection				Comparability	Outcome			NOS
	Representativeness of the exposed cohort	Selection of the non exposed cohort	Ascertainment of exposure	Demonstration that outcomes was not present at start of study	Comparability on the basis of the design or analysis	Assessment of outcome	Adequate follow-up duration	Adequate follow-up rate	Overall score
Xiao 2014 [28]	1	1	1	1	1	1	1	1	8
Tola 2017 [29]	0	1	1	1	1	1	1	1	7
GohariTaban 2019 [30]	1	1	1	1	1	1	1	1	8
GohariTaban 2021 [31]	1	1	1	1	1	1	1	1	8
Yang 2021 [32]	0	1	1	1	1	1	1	1	7

### Qualitative analysis

A study performed by Xiao et al., found that ADAMTS-1 level was positively correlated with the rates of oocyte maturity, oocyte recovery, and fertilization (*r* = 0.8313; *P* = 0.0403); the relationship between ADAMTS-1 levels and the rates of cleavage and good quality embryo, was not statistically significant [31]. Tola et al., found no significant association between ADAMTS-1 levels and metaphase II oocytes; however, ADAMTS-1 levels were positively correlated to implantation (data not shown) [32]. GohariTaban et al., found that ADAMTS-1 levels were positively correlated to oocyte recovery, oocyte maturation, and fertilization rates. Moreover, they pointed out that ADAMTS-9 levels were significantly correlated to oocyte recovery and oocyte maturation rates, while the relationship between ADAMTS-9 level and fertilization rate was not statistically significant [33]. GohariTaban et al., also found that ADAMTS-4 levels were positively correlated to oocyte recovery, oocyte maturation, and fertilization rates. Furthermore, there were significant associations between ADAMTS-5 level and oocyte recovery, oocyte maturation, and fertilization rates [34]. Yang et al., found that ADAMTS-1 levels were positively correlated to oocyte maturation and good-quality embryo rates; ADAMTS-1 levels were not associated with fertilization, cleavage, and blastocyst formation rates [35].

### Pooled Spearman coefficients

**Fig. 2:**
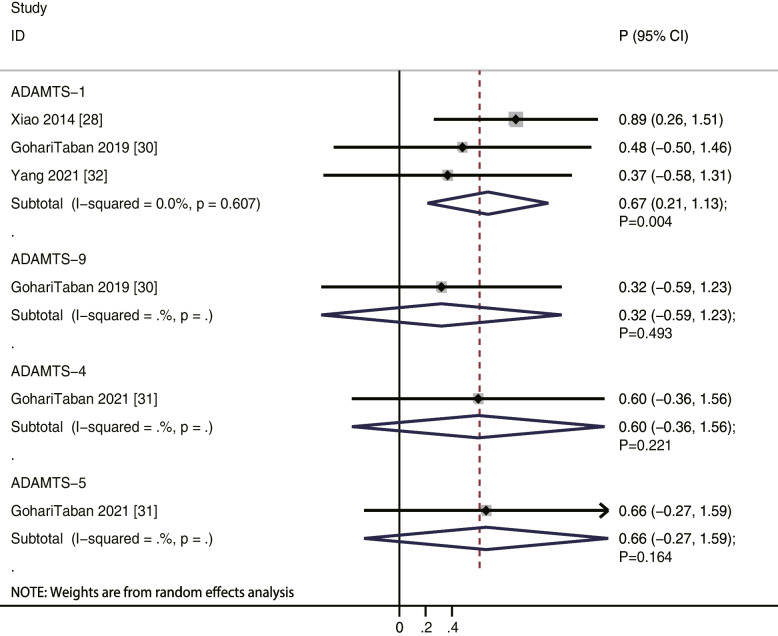
The relationship between ADAMTS family members and oocyte maturity rate

**Fig. 3:**
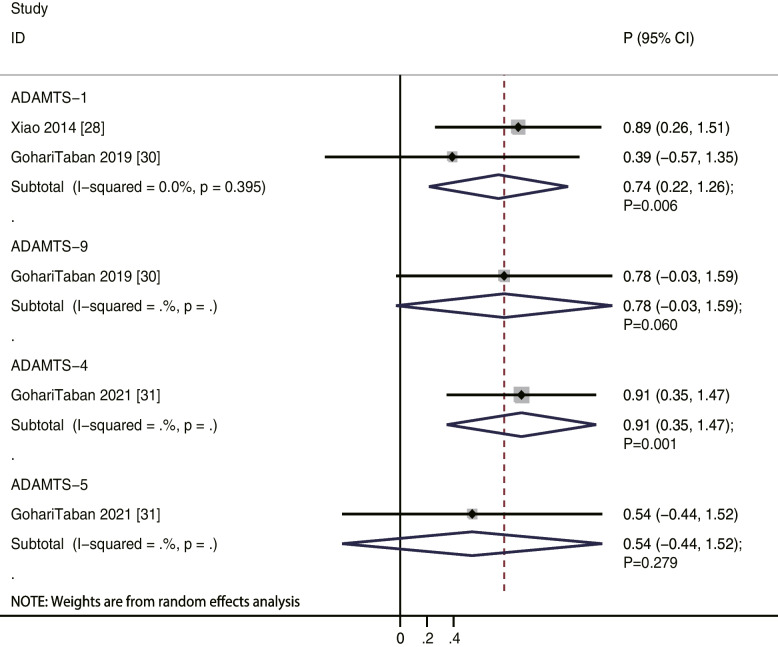
The relationship between ADAMTS family members and oocyte recovery rate

**Fig. 4:**
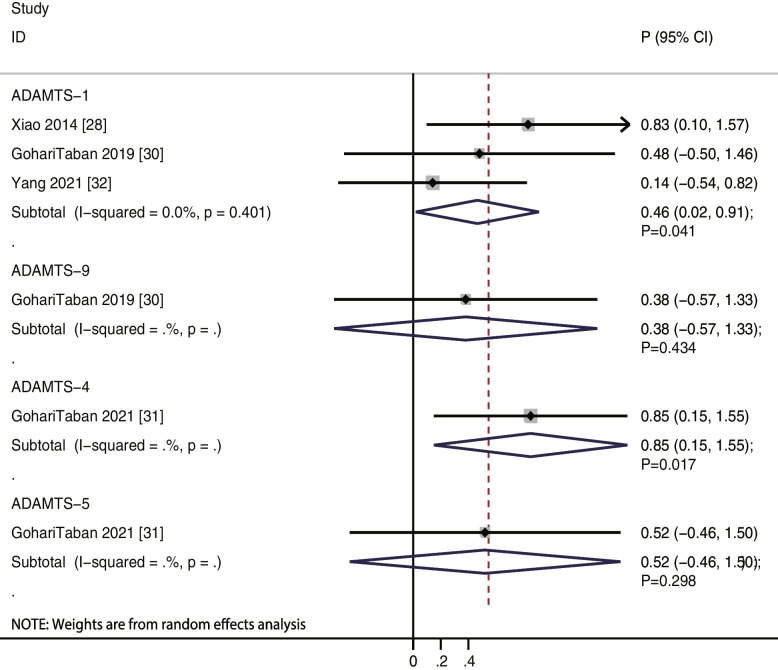
The relationship between ADAMTS family members and fertilization rate

**Fig. 5:**
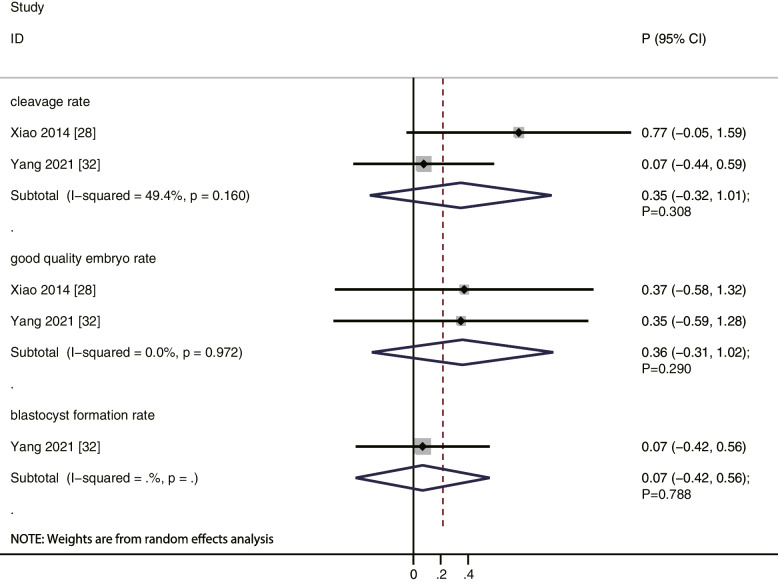
The relationship between ADAMTS-1 levels and cleavage, good-quality embryo, and blastocyst formation rates

We noted that ADAMTS-1 levels were positively correlated to oocyte maturity rate (*r* = 0.67; *P* = 0.004; without evidence of heterogeneity), while ADAMTS-4 (*r* = 0.60; *P* = 0.221), ADAMTS-5 (*r* = 0.66; *P* = 0.164), and ADAMTS-9 (*r* = 0.32; *P* = 0.493) levels were not (Fig. 2). Moreover, our results indicate that ADAMTS-1 (*r* = 0.74; *P* = 0.006) and ADAMTS-4 (*r* = 0.91; *P* = 0.001) levels were positively correlated to oocyte recovery rate, while ADAMTS-5 (*r* = 0.54; *P* = 0.279) and ADAMTS-9 (*r* = 0.78; *P* = 0.060) levels were not (Fig. 3). Similarly, ADAMTS-1 (*r* = 0.46; *P* = 0.041) and ADAMTS-4 (*r* = 0.85; *P* = 0.017) levels were significantly correlated to fertilization rate, while ADAMTS-5 (*r* = 0.52; *P* = 0.298) and ADAMTS-9 (*r* = 0.38; *P* = 0.434) levels were not (Fig. 4). Finally, the level of ADAMTS-1 was not associated with cleavage (*r* = 0.35; *P* = 0.308), good quality embryo (*r* = 0.36; *P* = 0.290), and blastocyst formation (*r* = 0.07; *P* = 0.788) rates (Fig. 5).

### Downregulation of ADAMTS family members and IVF-ET outcomes

A summary of the effects of downregulation of ADAMTS family members on IVF-ET outcomes is shown in Table 3. We noted that downregulation of ADAMTS-1, ADAMTS-4, ADAMTS-5, and ADAMTS-9 was associated with a large number of punctured follicles, retrieved, and Germinal vesicle oocytes, but did not affect Metaphase II oocytes and oocytes recovery, oocyte maturity, and fertilization rates. Moreover, ADAMTS-1 downregulation was not associated with cleavage, good-quality embryo, clinical pregnancy, and blastocyst formation rates.

**Table 3:** The pooled results for ADAMTS family members downregulation and IVF-ET Outcomes

Reported outcomes	ADAMTS family members	Number of studies	Effect estimates and 95%CI	*P* value	*I*^*2*^ (%)	Q statistic
Follicles punctured	ADAMTS-1	2	7.24 (4.61 to 9.88)	< 0.001	0.0	0.374
	ADAMTS-4	1	7.20 (3.69 to 10.71)	< 0.001	-	-
	ADAMTS-5	1	7.20 (3.69 to 10.71)	< 0.001	-	-
	ADAMTS-9	1	6.38 (3.13 to 9.63)	< 0.001	-	-
Oocytes retrieved	ADAMTS-1	3	1.61 (1.28 to 1.93)	< 0.001	0.0	0.379
	ADAMTS-4	1	3.63 (1.19 to 6.07)	0.004	-	-
	ADAMTS-5	1	3.63 (1.19 to 6.07)	0.004	-	-
	ADAMTS-9	1	3.20 (0.90 to 5.50)	0.006	-	-
Metaphase II oocytes	ADAMTS-1	2	1.14 (-1.74 to 4.02)	0.439	0.0	0.958
	ADAMTS-4	1	1.30 (-0.64 to 3.24)	0.189	-	-
	ADAMTS-5	1	1.30 (-0.64 to 3.24)	0.189	-	-
	ADAMTS-9	1	1.30 (-5.47 to 8.07)	0.707	-	-
Germinal vesicle oocytes	ADAMTS-1	1	2.89 (2.64 to 3.14)	< 0.001	-	-
	ADAMTS-4	1	2.19 (2.01 to 2.37)	< 0.001	-	-
	ADAMTS-5	1	2.19 (2.01 to 2.37)	< 0.001	-	-
	ADAMTS-9	1	2.89 (2.64 to 3.14)	< 0.001	-	-
Oocytes recovery rate	ADAMTS-1	2	0.27 (0.05 to 1.61)	0.152	40.6	0.195
	ADAMTS-4	1	0.08 (0.00 to 1.47)	0.089	-	-
	ADAMTS-5	1	0.08 (0.00 to 1.47)	0.089	-	-
	ADAMTS-9	1	0.06 (0.00 to 1.19)	0.066	-	-
Oocyte maturity rate	ADAMTS-1	3	0.64 (0.32 to 1.29)	0.211	0.0	0.900
	ADAMTS-4	1	0.63 (0.21 to 1.89)	0.405	-	-
	ADAMTS-5	1	0.63 (0.21 to 1.89)	0.405	-	-
	ADAMTS-9	1	0.52 (0.17 to 1.63)	0.263	-	-
Fertilization rate	ADAMTS-1	3	0.85 (0.50 to 1.45)	0.554	0.0	0.498
	ADAMTS-4	1	0.63 (0.25 to 1.62)	0.340	-	-
	ADAMTS-5	1	0.63 (0.25 to 1.62)	0.340	-	-
	ADAMTS-9	1	0.90 (0.36 to 2.24)	0.815	-	-
Cleavage rate	ADAMTS-1	2	0.33 (0.05 to 2.15)	0.246	0.0	0.954
Good-quality embryos rate	ADAMTS-1	2	0.76 (0.41 to 1.41)	0.381	0.0	0.586
Blastocyst formation rate	ADAMTS-1	1	0.68 (0.30 to 1.57)	0.371	-	-
Clinical pregnancy rate	ADAMTS-1	1	0.90 (0.37 to 2.20)	0.820	-	-

## Discussion

This study is the first to assess the role of ADAMTS family members in the outcomes of IVF-ET for patients with PCOS, using the meta-analytic approach. A total of 181 patients with PCOS from 5 studies were included, along with a wide range of patient characteristics. This study found that the oocyte maturity, oocyte recovery, and fertilization rates, were affected by ADAMTS-1 levels. Moreover, ADAMTS-4 levels were positively correlated to oocyte recovery and fertilization rates. Finally, the downregulation of ADAMTS-1, ADAMTS-4, ADAMTS-5, and ADAMTS-9 was associated with elevated follicle puncture, oocytes retrieval, and Germinal vesicle oocytes levels.

The methodology of included studies was systematically assessed, and all included studies were of high quality. The cutoff value of the ADAMTS family members in patients with PCOS varied across the included studies, while could affect the net effect estimates for the role of ADAMTS family members. Moreover, 2 of the included studies absent the representativeness of the cohort, and several members of the ADAMTS family were obtained from only a few of the included studies; therefore, the results are not reliable. Therefore, the results of this study should be cautiously applied as they require verification by further large-scale prospective studies.

We noted that ADAMTS-1 levels were positively correlated to oocyte maturity, oocyte recovery, and fertilization rates and that downregulation of ADAMTS-1 was significantly correlated to elevated follicle puncture, oocytes retrieval, and Germinal vesicle oocytes levels. A possible reason for this could be the ADAMTS-1 mainly expressed in the granulosa cells of mammalian preovulatory follicles, which could induced by LH through transactivation of the PG receptor, and suggested ADAMTS-1 play an important role in ovulation and folliculogenesis [17, 19, 36]. Moreover, ADAMTS-1 modulates cell signaling and could affect the sequestration of signaling factors within the cumulus/oocyte microenvironment by physical or oxidative stress through versican cleavage; this could be useful in predicting oocyte capacity and subsequent pregnancy [37–39].

This study found the oocyte recovery and fertilization rates to be significantly correlated to ADAMTS-4 levels, and ADAMTS-4 downregulation to be significantly correlated to elevated follicle puncture, oocytes retrieval, and Germinal vesicle oocytes levels. Moreover, the follicle puncture, oocytes retrieval, and Germinal vesicle oocytes levels were affected by ADAMTS-5, and ADAMTS-9 downregulation. ADAMTS-1, ADAMTS-4, and ADAMTS-5 have overlapping effects on aggrecan, versican, and brevican degradation [40]. Altered levels of ADAMTS-1, ADAMTS-4, and ADAMTS-5 could affect the main component of the cumulus-oocyte complex, and the cumulus cells gene expression was regarded as a most promising oocyte quality marker [32, 41, 42]. Moreover, ADAMTS-9 is involved in extracellular matrix binding and expression during embryogenesis, and ADAMTS-9 downregulation in the cumulus cells is significantly correlated to oocytes maturation arrest [43, 44].

Although ADAMTS-1 levels were positively correlated to oocyte maturity, oocyte recovery, and fertilization rates, ADAMTS-4 levels were only positively correlated to oocyte recovery and fertilization rates. The downregulation of ADAMTS-1 and ADAMTS-4 was not associated with the oocyte recovery, oocyte maturity, fertilization, cleavage, good-quality embryo, clinical pregnancy, and blastocyst formation rates. The possible reason for this could be the small number of studies that reported these outcomes, and the power might not be enough to detect the potential role of ADAMTS family members in IVF-ET outcomes.

Owing to the PCOS could affect oocyte competence and endometrial function, and causing series pregnancy complications [12–14], the prognostic factor for IVF-ET outcomes in patients with PCOS should be explored. This study found IVF-EF outcomes could be affected by ADAMTS-1 and ADAMTS-4 in patients with PCOS. Therefore, the ADAMTS for PCOS patients should be screened for patients at high risk, then the intervention should be performed to improve further IVF-ET outcomes.

This study has several limitations: (1) the analysis included both prospective and retrospective studies, and there could be selection and recall biases; (2) the background therapies for PCOS varied across the included studies; this could affect the role of ADAMTS family members; (3) the role of ADAMTS-4, ADAMTS-5, and ADAMTS-9 in IVF-ET outcomes was discussed in just a few studies, and several other outcomes were not investigated, such as implantation, abortion, miscarriage, and repeat implantation failure; (4) the relationship between ADAMTS family members and IVF-ET outcomes based on univariate regression, and the characteristics of patients, were not adjusted; (5) subgroup analysis were not performed owing to smaller number of included studies; and (6) inherent limitations of meta-analysis in the published articles, including restricted detailed analyses and inevitable publication bias.

## Conclusions

Our study found that ADAMTS-1 levels were positively correlated to oocyte maturity, oocyte recovery, and fertilization rates, while ADAMTS-4 levels were positively correlated to oocyte maturity, oocyte recovery, and fertilization rates. Moreover, the downregulation of ADAMTS-1, ADAMTS-4, ADAMTS-5, and ADAMTS-9 was significantly correlated to elevated follicle puncture, oocytes retrieval, and Germinal vesicle oocytes levels. Further large-scale prospective studies should be conducted to verify the results of this study and compare the role of ADAMTS downregulation on implantation, abortion, miscarriage, repeat implantation failure.

## Data Availability

The datasets used and/or analysed during the current study are available from the corresponding author on reasonable request.

## Abbreviations

ADAMTS: A disintegrin and metalloproteinase with thrombospondin- like motifs
BMI: Body mass index
IVF-ET: In vitro fertilization and embryo transfer
NOS: Newcastle–Ottawa Scale
PCOS: Polycystic ovary syndrome
PRISMA: Preferred reporting items for systematic reviews and meta-analysis
